# Combining Load and Motor Encoders to Compensate Nonlinear Disturbances for High Precision Tracking Control of Gear-Driven Gimbal

**DOI:** 10.3390/s18030754

**Published:** 2018-03-02

**Authors:** Tao Tang, Sisi Chen, Xuanlin Huang, Tao Yang, Bo Qi

**Affiliations:** 1 Institute of Optics and Electronics, Chinese Academy of Science, Chengdu 610209, China; chensisi13@yeah.net (S.C.); huang3xuanlin@163.com (X.H.); Yangtao@gmail.com (T.Y.); qibo@ioe.ac.cn (B.Q.); 2 Key Laboratory of Optical Engineering, Chinese Academy of Sciences, Chengdu 610209, China; 3 Department of Mechanical engineering, University of Chinese Academy of Sciences, Beijing 100039, China

**Keywords:** sensor-fusion, nonlinear disturbances, rate deviation, DOB, gear-driven

## Abstract

High-performance position control can be improved by the compensation of disturbances for a gear-driven control system. This paper presents a mode-free disturbance observer (DOB) based on sensor-fusion to reduce some errors related disturbances for a gear-driven gimbal. This DOB uses the rate deviation to detect disturbances for implementation of a high-gain compensator. In comparison with the angular position signal the rate deviation between load and motor can exhibits the disturbances exiting in the gear-driven gimbal quickly. Due to high bandwidth of the motor rate closed loop, the inverse model of the plant is not necessary to implement DOB. Besides, this DOB requires neither complex modeling of plant nor the use of additive sensors. Without rate sensors providing angular rate, the rate deviation is easily detected by encoders mounted on the side of motor and load, respectively. Extensive experiments are provided to demonstrate the benefits of the proposed algorithm.

## 1. Introduction

Due to the practical issues, such as size and power, which are associated with the increase of the motor capacity, more and more designers tend to seek for the designs with gear attached motors [1,2]. Thus, low power DC motor coupled with gear-box to obtain high torque is well adopted in modern servo systems. However, it is inevitable that the gear-driven control also introduces some new problems to the systems, such as backlash [3,4,5], flexibility [6], hysteresis [7], and nonlinear friction [8]. The friction and backlash are the most common non-smooth nonlinearities that may degrade the control performance [9,10]. Backlash occurs due to the gap existing between the inner motor axis and the outer load axis with gear box used to connect. As a result, the motion transmission is affected by the spacing of the gear. Especially, when reciprocating movement happens in the gear-driven control system, the position precision could deteriorates, and even the closed-loop system may fall into limit cycle [10], which could lead to the closed-loop control system unstable. In fact, reciprocating movement always occurs in a tracking control system of gimbal, because target trajectory can move forth and back or down and up. An approach based on dual motors connected in parallel to the load to eliminate backlash without by means of software and feedback control [11]. But, these mechanical techniques require changing or adding some parts on the mechanical systems, leading to high production cost. Therefore, advanced control techniques are necessary to compensate these nonlinear disturbances. Methods of Model-based [12,13,14] control to eliminate these disturbances are developed by some authors, but these techniques are deeply dependent on an accurate model. Sensor-less backlash compensation is very interesting [15]. However, at the motion reversal, the gap due to non-contaction will cause undesirable effects on position control. Adaptive control [16] based on an estimation of the disturbing torque is developed, but a good choice of adaptive control parameters will lead to a good result. The DOB techniques could be very attractive to cope with disturbances, but the DOB is based on the inverse model of the plant, combined with Q-filter to estimate the torque disturbance [17,18].

We present a mode-free DOB based on sensor-fusion control to improve closed-loop performance of position control for a gear-driven gimbal. This proposed method uses the rate deviation to detect disturbances for implementing this DOB, which can be plugged into the originally feedback control mode. The rate deviation between load and motor can exhibits the disturbances exiting in the gear-driven gimbal quickly when compared with position signal. Without rate sensors providing angular rate of the gimbal, the rate deviation is easily detected by encoders mounting on the side of motor and load respectively. Due to high bandwidth of the motor rate closed loop, the inverse model of the plant is not necessary. Besides, this DOB does not require either complex modeling of a plant or the use of additive sensors. This paper gives an example of two-axis gear-driven gimbal [19] to testify our proposed control method. The remainder is organized as follow: Section 2 presents a detailed introduction to control model of gear-driven gimbal, mainly describing control model. Section 3 discusses and analyzes system robustness. Section 4 focuses on the implementation of the proposed DOB. Section 5 sets up simulations and experiments to testify the theorems above. Concluding remarks are presented in Section 6.

## 2. Control Model of Gear-Driven Gimbal

**Figure 1:**
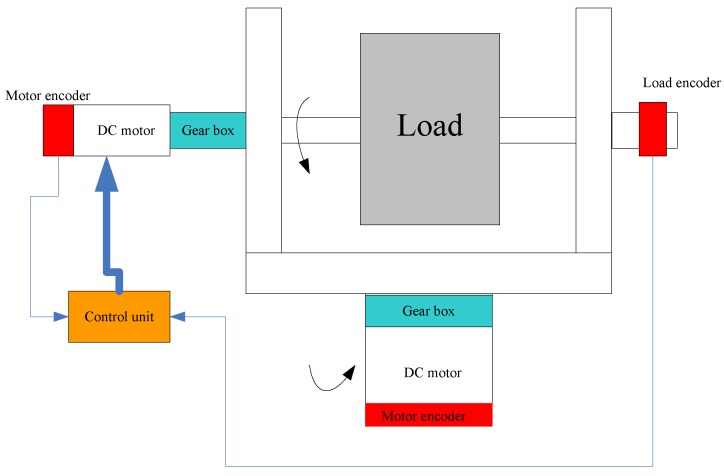
The layout of gear-driven system.

A two-axis of gear-drive system is depicted, as seen in Figure 1. It is made of DC motor, gear box reducer, load, and control unit. In a modern control system, the angular position and velocity can be provided by encoder. Due to existing transmission flexibility between the load and the motor, encoders are mounted in the load side and in the motor side, respectively, to provide the rate deviation, which can be used to detect disturbances. such as backlash, friction, and unmodelled dynamics. Due to the symmetry, the elevation axis is considered as an example to verify the proposed control method in this paper.

**Figure 2:**
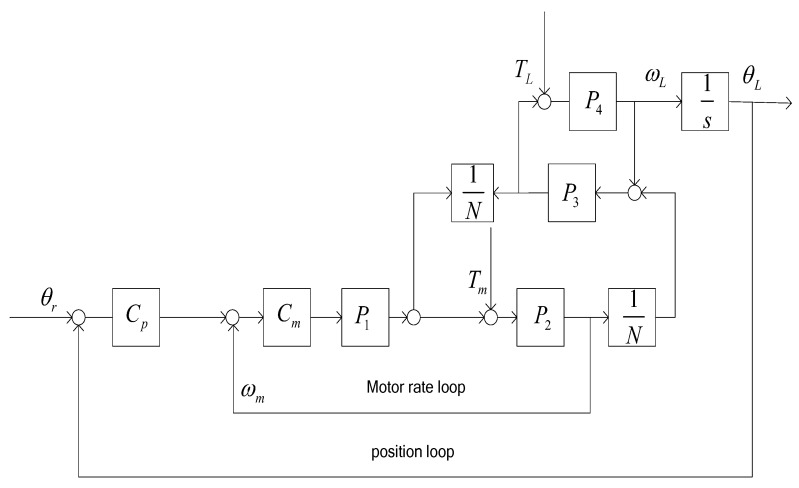
The classical control block diagram of gear-drive gimbal. $C_{p}(s)$: position controller; $C_{m}(s)$: speed loop controller; $\omega_{L}(s)$: load angular velocity; $\omega_{m}(s)$: motor angular velocity; $\theta_{L}(s)$: load angular position; $\theta_{r}(s)$: reference angular position; $P_{1}(s)$: torque constant of motor; $P_{2}(s)$: mechanical transfer function of motor; N: reduction ratio; $P_{3}(s)$: the transfer function of gear; $P_{4}(s)$: load transfer function; $T_{L}(s)$: disturbance forces in the load side; $T_{m}(s)$: disturbance forces in the motor side.

The block diagram of gear-driven control system structure can be depicted in Figure 2. The classical control mode includes the motor rate loop and the load position loop.

## 3. Robustness Analysis of the Rate Loop

**Figure 3:**
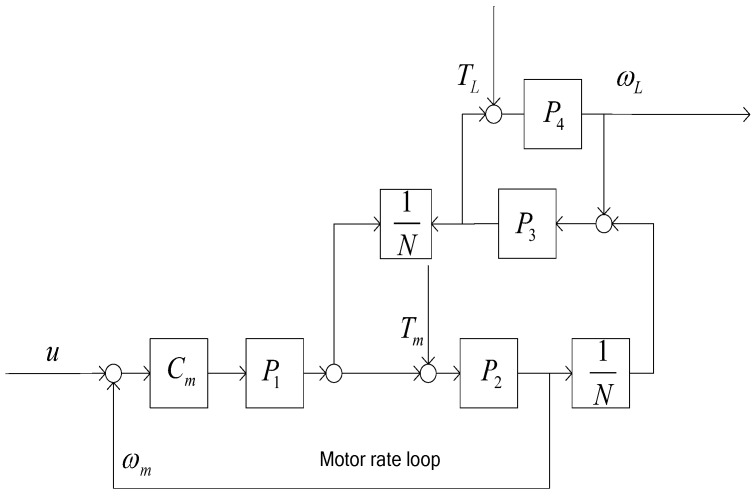
The control block diagram with the motor rate loop.

With only the rate closed loop in motor side shown in Figure 3, the transfer function $\omega_{L}(s)$ can be represented as follows:

(1) $$\begin{array}{l} {\omega^{\prime }}_{L}=\frac{C_{m}P_{1}P_{2}P_{3}P_{4}N}{N^{2}+P_{3}P_{4}N^{2}+P_{2}P_{3}+C_{m}P_{1}P_{2}P_{3}P_{4}N^{2}+C_{m}P_{1}P_{2}N^{2}}u\\ +\frac{P_{2}P_{3}P_{4}N}{N^{2}+P_{3}P_{4}N^{2}+P_{2}P_{3}+C_{m}P_{1}P_{2}P_{3}P_{4}N^{2}+C_{m}P_{1}P_{2}N^{2}}T_{m}\\ +\frac{P_{4}N^{2}+P_{1}P_{2}P_{4}N^{2}C_{m}+P_{2}P_{3}P_{4}}{N^{2}+P_{3}P_{4}N^{2}+P_{2}P_{3}+C_{m}P_{1}P_{2}P_{3}P_{4}N^{2}+C_{m}P_{1}P_{2}N^{2}}T_{L} \end{array}$$

The sensitive expression will explain that whether the performance of the control system will change or not, when one of system’s characteristics is changed. The sensitive expression defined by Horowitz is shown below:

(2) $$S_{k}^{\phi }=\frac{\Delta \phi (s)/\phi (s)}{\Delta k(s)/k(s)}=\frac{\phi^{\prime }(s)-\phi (s)}{k^{\prime }(s)-k(s)}\frac{k(s)}{\phi (s)}$$

Here, it assumes that the characteristic of motor is changed. Namely, $P_{2}$ is replaced by ${P^{\prime }}_{2}$. We easily get the below equations

(3) $$M_{1}=\left(\frac{{\omega^{\prime }}_{L}}{u}\right)=\frac{C_{m}P_{1}P_{2}P_{3}P_{4}N}{N^{2}+P_{3}P_{4}N^{2}+P_{2}P_{3}+C_{m}P_{1}P_{2}P_{3}P_{4}N^{2}+C_{m}P_{1}P_{2}N^{2}},$$

(4) $${M^{\prime }}_{1}=\left(\frac{{\omega^{\prime }}_{L}}{u}\right)=\frac{C_{m}P_{1}{P^{\prime }}_{2}P_{3}P_{4}N}{N^{2}+P_{3}P_{4}N^{2}+{P^{\prime }}_{2}P_{3}+C_{m}P_{1}{P^{\prime }}_{2}P_{3}P_{4}N^{2}+C_{m}P_{1}{P^{\prime }}_{2}N^{2}}.$$

Substituting (3) and (4) into (2), yielding

(5) $$S_{{P^{\prime }}_{2}}^{M_{1}}=\frac{1}{1+\frac{{P^{\prime }}_{2}P_{3}}{N^{2}+P_{3}P_{4}N^{2}}+C_{m}P_{1}{P^{\prime }}_{2}}.$$

When considering $\frac{{P^{\prime }}_{2}P_{3}}{N^{2}+P_{3}P_{4}N^{2}}\approx 0$ because of N>>1, we have $S_{{P^{\prime }}_{2}}^{M_{1}}=\frac{1}{1+C_{m}P_{1}{P^{\prime }}_{2}}$. $\left|C_{m}P_{1}P^{\prime }\right|\geq 1$ is required if the change of motor feature can be neglected. In fact, $C_{m}P_{1}P^{\prime }$ is considered as the rate open-loop transfer function of motor and its magnitude response is located above the 0 dB below the cutoff frequency.

Based on the above analysis, we have $\omega_{L}/T_{m}\approx 0$, and

(6) $$\frac{{\omega^{\prime }}_{L}}{T_{L}}\approx \frac{P_{4}+P_{1}P_{2}P_{4}C_{m}}{1+P_{3}P_{4}+C_{m}P_{1}P_{2}P_{3}P_{4}+C_{m}P_{1}P_{2}}$$

Considering $C_{m}P_{1}P$ has high gain in the low frequencies and approaches to zero in the high frequencies. The (6) is rewritten into

(7) $$\frac{{\omega^{\prime }}_{L}}{T_{L}}\approx \frac{P_{4}}{1+P_{3}P_{4}}$$

As we can see from (7), ${\omega^{\prime }}_{L}/T_{L}\approx 1/2$, is reasonable in the low frequency. Thus, the motor rate loop contributes finitely to rejecting the load disturbance. In gear-drive control system, one of the important problems is to suppress the nonlinear disturbances induced by gear box in the load side. When adding the rate closed loop in the load side, the load rate is expressed below,

(8) $$\omega_{L}=\frac{C_{L}M_{1}}{1+C_{L}M_{1}}u+\frac{{\omega^{\prime }}_{L}/T_{m}}{1+C_{L}M_{1}}T_{m}+\frac{{\omega^{\prime }}_{L}/T_{L}}{1+C_{L}M_{1}}T_{L}$$

In the same way, the load rate loop can improve the closed-loop performance for the input u and further reduces the effect of disturbances in the motor side, but it has a limitation of rejecting the load disturbances because the open-loop gain of $C_{L}M_{1}$ must be restrained by the mechanical resonances of $P_{4}$.

## 4. Disturbance Observer of the Gear-Drive Gimbal

**Figure 4:**
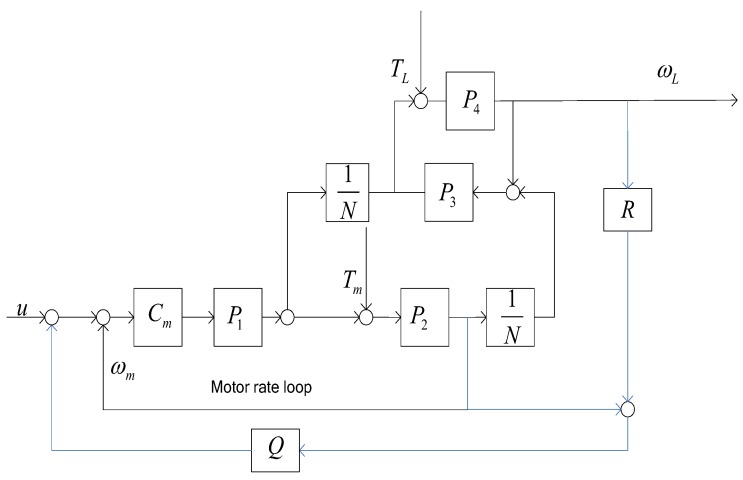
Disturbance observer (DOB) of the gear-drive gimbal.

Note that the disturbances in the load side cannot be well mitigated by the motor rate loop with or without the load rate loop. In this section we introduce a DOB based on the rate deviation to reject disturbances, which is depicted in Figure 4.

**Figure 5:**
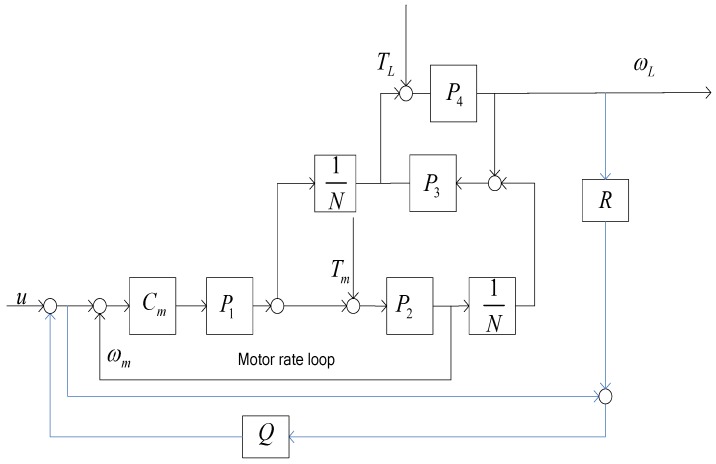
The proposed Disturbance observer.

The rate deviation between the motor side and the load side is used to implement the DOB, resulting in disturbance rejection. However, it is very complicate to model the transfer function from u to $\omega_{L}$ such that the DOB is difficult to design for meeting the intention. In fact, the motor rate closed loop makes the input nearly equal to the output. Therefore, a proposed control structure is given in Figure 5.

The load rate with DOB plugging into the motor rate loop is shown in Figure 5 derived below

(9) $$\omega_{L}=\frac{M_{1}}{1-Q+QM_{1}R}u+\frac{{\omega^{\prime }}_{L}/T_{m}(1-Q)}{1-Q+QM_{1}R}T_{m}+\frac{{\omega^{\prime }}_{L}/T_{L}(1-Q)}{1-Q+QM_{1}R}T_{L}$$

How to design Q and R is the first step. The perfect condition of canceling disturbances is 1−Q=0 under the condition of the perfect control stability $-Q+QM_{1}R=0$. Therefore, $M_{1}$ plays an important role for the parameters design. The (3) is rewritten into below

(10) $$M_{1}=\frac{1}{N}\frac{C_{m}P_{1}P_{2}}{1+C_{m}P_{1}P_{2}}\frac{P_{3}P_{4}}{1+\frac{C_{m}P_{1}P_{2}}{1+C_{m}P_{1}P_{2}}P_{3}P_{4}+\frac{P_{2}P_{3}}{N^{2}}}$$

When considering N>>1 and $\frac{C_{m}P_{1}P_{2}}{1+C_{m}P_{1}P_{2}}\approx 1$, the (10) is simplified, as follows

(11) $$M_{1}=\frac{1}{N}\frac{P_{3}P_{4}}{1+P_{3}P_{4}}$$

For the DOB not affecting the closed-loop stability, the perfect condition is required

(12) $$R=M_{1}^{-1}=N(1+\frac{1}{P_{3}P_{4}})$$

Due to either $P_{3}(s)$ or $P_{4}(s)$ all being the second-order low-pass filter, $P_{3}P_{4}\approx 1$ is reasonable in the low frequency. The Q-filter design is guided by below

(13) $$1-Q+QM_{1}R=1$$

Substituting R=2N into (13), we have

(14) $$Q\frac{1-P_{3}P_{4}}{1+P_{3}P_{4}}=0$$

It is impossible to arrive at $-Q+QM_{1}R=0$. So, the bandwidth of Q needs to be less than that of $\frac{1-P_{3}P_{4}}{1+P_{3}P_{4}}$.

## 5. Experimental Setup

**Figure 6:**
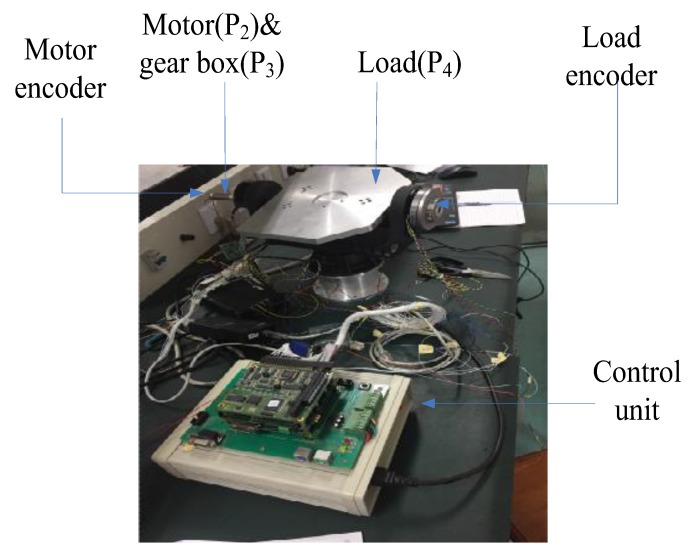
Experimental configuration of the gear-driven gimbal.

The two-axis gear-driven gimbal is shown in Figure 6. Two optical encoders are installed in the two-axis gimbals to measure the angular position, and also can provide gimbal velocity through encoder difference. The control units include mainly Analog Digital Converter (A/D), Digital Analog Converter (D/A), computer (PC104), and Field Programmable Gate Array (FPGA). The motor is a high speed brush DC motor. The absolute-type rotary encoder is installed at the motor side. Its linear numbers are 1024 counts/round. The reduction ratio of the gear is 1227. The load encoder is 21-bit, and has a resolution of is $0.618^{\prime \prime }$. The digital signals of optical encoders are transmitted into the computer through serial port communication. The PC104 uses these sensor signals to implement controller for activating the power driving amplification to control the Gimbal.

**Figure 7:**
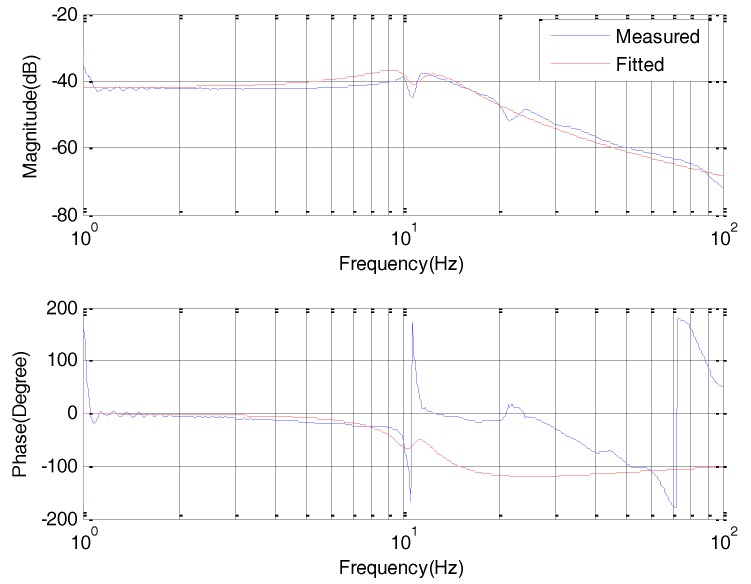
Bode response of $M_{1}$.

A Bode Plot based on Fourier Transform Theorem is a useful tool that shows the gain and phase response of a given control system for different frequencies, which describes the control system straightforward. The motor rate closed-loop response is shown by Figure 7. In fact, this response is measured from u. to $w_{L}$, because the load encoder has enough precision to provide more precise velocity than that with the motor encoder. The closed-loop response can provide design criteria for the position controller in Figure 2 and the DOB controller in Figure 5.

A $Q_{31}$-filter [20,21] is considered as the robust Q-filter shown below

(15) $$Q=\frac{3as+1}{(as+1){}^{3}}$$.

Substituting the fitting transfer function in Figure 7 in to (13), an optimal $Q_{31}$-filter is chosen, resulting in the rate response with the DOB based on the motor rate loop in Figure 7.

**Figure 8:**
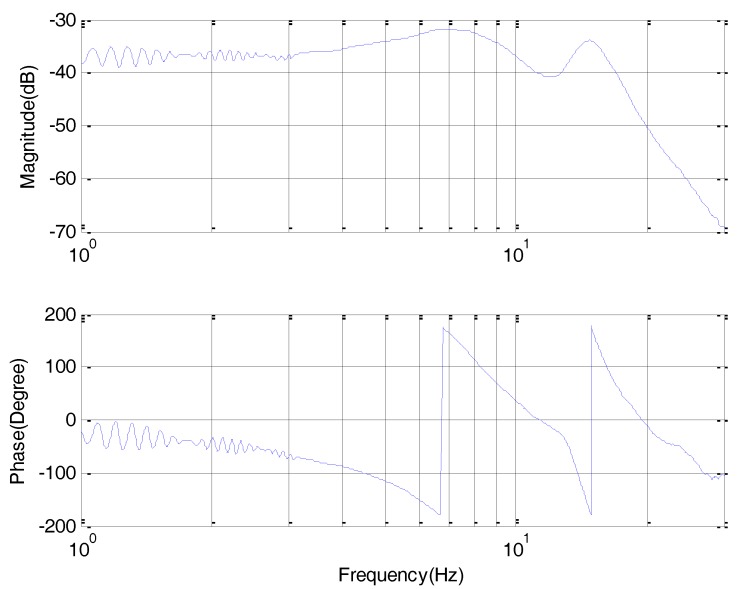
Rate Bode response with DOB.

In comparison with the motor closed-loop response in Figure 7, the bandwidth shown in Figure 8 becomes narrow for assuring the control stability with a low bandwidth of $Q_{31}$-filter.

**Figure 9:**
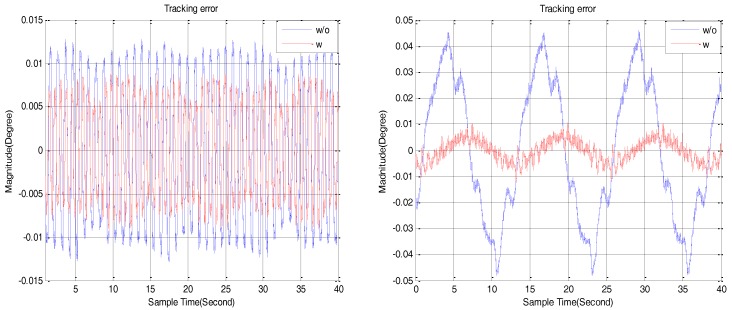
Tracking error under different reference angular positions. The left reference angular position is the sinusoidal trajectory of *θ_r_* = 0.01°sin0.5*t*, and the right is the sinusoidal trajectory of *θ_r_* = 5°sin0.5*t*.

For verifying the proposed method to be effective, experiments regarding the trajectory track are demonstrated. The resulting errors with the position loop closed are exhibited in Figure 9. When the reference angular position is *θ_r_* = 0.01°sin0.5*t*, the improved DOB can keep the steady state error lower than 0.01 degree, when approximated to the encoder precision. Without the DOB, disturbances regarding the friction and backlash lead to dead zone, which can be seen obviously in Figure 9 if the reference angular position is *θ_r_* = 5°sin0.5*t*. The DOB based on the sensor-fusion makes the tracking error smaller than that without the DOB both in the low-velocity tracking and the high-velocity tracking.

## 6. Conclusions

A mode-free DOB based on sensor fusion for a gear-driven gimbal is proposed to reduce some error related disturbances. The DOB can be plugged into the originally control mode, which can bring a high gain to the closed-loop system. Complex modeling of disturbances and the use of additive sensors are not needed. In this paper, we focus on the implementation of the DOB, the optimization of the control parameters, and the analysis of the close-loop stability. Experimental results show the control method of the DOB has great performance to eliminate the nonlinear disturbances and reduce the turning error. In comparison with the originally two closed loops, the tracking error with the proposed control mode has a reduction by a factor of three. In the future work, the application of AI methods (evolutionary algorithms and neural networks) to develop the model for control application is promising to further improve closed-loop system [22,23,24].
